# Pair-bonding on the brain

**DOI:** 10.1038/s42003-023-05711-3

**Published:** 2024-01-09

**Authors:** Lindsey T. Thurston

**Affiliations:** Department of Psychology, University of Toronto, Toronto, Canada

## Abstract

As a monogamous species, the prairie vole is a common model for social neuroscience. Gustison and colleagues mapped a whole-brain histological atlas of the prairie vole and used this atlas to identify a neural network of pair-bonding behaviour. The study reveals coordinated neural networks in mated pairs and highlights the influence of social bonding on neural processing in the adult prairie vole brain.

**Figure:**
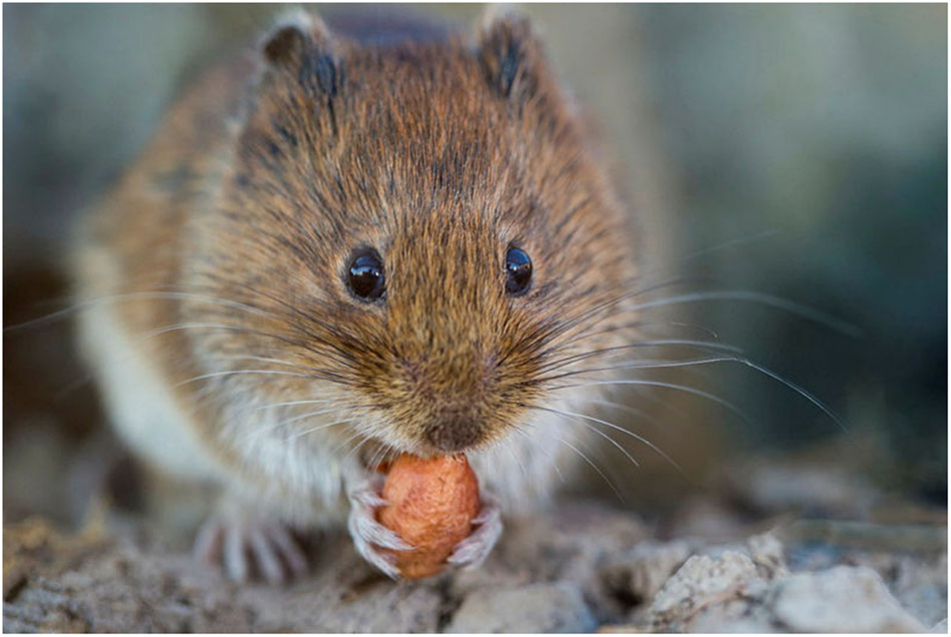
HPE, stock.adobe.com

At least for the monogamous prairie vole, differences in sexual behaviour between males and females may not be associated with sexually dimorphic brain networks. Animal sexual behaviour as a whole is characterized as sexually dimorphic behaviour. For example, female voles hold a lordosis position where the back is arched and the pelvis is raised in the air, while males perform mounting behaviours^1^. Despite these behavioural differences, a recently identified neural network associated with pair bonding and mating in prairie voles demonstrates coordinated connectivity between opposite-sex mates^2^, rather than sexually dimorphic or distinct neural networks.

A research group from the University of Texas at Austin investigated the neural networks of pair bonding in the prairie vole brain, as described in their peer-reviewed preprint in *eLife*^2^. Opposite-sex prairie vole pairs and same-sex cage mates were observed at one of four bonding stages over a 22 h period: acclimation, initial mating, initial bond formation, or bond stabilization. The observed behaviours were scored manually for paired (e.g., mounting) or individual behaviours (e.g., self-grooming) and then associated with neural activity by using whole-brain c-Fos immunostaining, a method that distinguishes recently active neurons by the presence of the c-Fos protein.

As a result, Gustison and colleagues^2^ identified an extensive network of regions within the hypothalamus and amygdala that were active during mating and bonding stages. Unexpectedly, no sex-specific connections were found, and instead mated pairs had a coordinated change in neural activation. In fact, ejaculation, an exclusively male behaviour in prairie voles, was strongly related to the pair-bonding neural activity in both males and females. This means that the rate of male ejaculation influenced neural activity patterns in females in the same way that it influenced the male brain. These findings join other examples of neural activity that is coupled between socially interacting individuals, including non-sexual or same-sex pairings, such as in mice and primates like humans^3,4^.

While previous studies have demonstrated that brain-to-brain coupling is integral to non-sexual socialization activities such as communication and signal perception^4^, the convergence of male and female neural activation during pair-bonding renews questions of sex-related differences in the brain. Prairie voles are a popular model for studying the social brain because of their monogamous mating strategy. Yet, translation to human experiences is limited as human sexual behaviour boasts greater variation. Future research should consider social coupling in non-monogamous and same-sex romantic pairings to broaden our understanding of pair-bonding on the brain.
